# Molecular Characterization and Phylogenetic Analysis of* Listeria monocytogenes* Isolated from Milk and Milk Products in Kaduna, Nigeria

**DOI:** 10.1155/2016/4313827

**Published:** 2016-08-11

**Authors:** U. B. Usman, J. K. P. Kwaga, J. Kabir, O. S. Olonitola, S. Radu, F. Bande

**Affiliations:** ^1^Department of Veterinary Services, Ministry of Animal Health and Fisheries Development, Usman Faruk Secretariat, Sokoto, Sokoto State 840221, Nigeria; ^2^Department of Veterinary Public Health and Preventive Medicine, Faculty of Veterinary Medicine, Ahmadu Bello University, Zaria 810004, Nigeria; ^3^Department of Microbiology, Faculty of Science, Ahmadu Bello University, Zaria 810004, Nigeria; ^4^Center of Excellence for Food Safety Research, Faculty of Food Science and Technology, Universiti Putra Malaysia, 43400 Serdang, Selangor Darul Ehsan, Malaysia; ^5^Department of Pathology and Microbiology, Faculty of Veterinary Medicine, Universiti Putra Malaysia, 43400 Serdang, Selangor Darul Ehsan, Malaysia

## Abstract

In this study,* Listeria* (*L.*)* monocytogenes* isolated from milk and milk products in Kaduna, Nigeria, were subjected to a multiplex PCR assay to identify virulence-associated genes (such as* prf* A,* inl* A,* hly* A,* act* A, and* iap*). Of the 36 isolates, 9 (25%) were positive for one or two virulence-associated genes. Based on the sample type, 6 (16.9%) of the isolates that possessed virulence-associated genes were obtained from raw milk, 2 (3.2%) from “Manshanu,” and 1 (2.8%) from “Kindrimo.” Sequence and phylogenetic analysis based on the 16S rRNA revealed that Nigerian* L. monocytogenes* isolates (NGA 34A, NGA 35A, NGA 41A, and NGA 38A), when compared with reference* L. monocytogenes*, were grouped into two distinct clusters, A and B, with sequence (NGA 34A, NGA 35A, and NGA 41A) phylogenetically closer to J1776; N1-011A; R2-502; J1816; and J2-031, whereas* L. monocytogenes* isolate (NGA 38A) clustered with EDG; J1-220; J1926; J1817; and J2-1091. The separation of the Nigerian* L. monocytogenes* isolates into linage A (responsible for epidemic listeriosis) and lineage B (responsible for sporadic cases of listeriosis) is of public health concern and that local isolates might have potentials for human food borne listeriosis based on the virulence factors so far identified.

## 1. Introduction

Listeriosis is a food borne infection caused by opportunistic bacterial pathogen* Listeria (L.) monocytogenes* that is abundant in the environment [1]. The bacteria have been isolated from the soil, water, plants, faeces, decaying vegetables, meat, sea food, dairy products, and asymptomatic human and animal carriers [2]. It has been isolated from sheep, goat, and cow milk [3].* L. monocytogenes* is a Gram-positive facultative intracellular food borne pathogen causing listeriosis, a rare but severe infection in humans and animals with a mortality rate of 25–30% [4]. Listeriosis is either noninvasive, self-limiting, and gastrointestinal, occurring in healthy individuals, or invasive and systemic, occurring in immunocompromised individuals such as pregnant women and newborns, resulting in meningitis, encephalitis, septicaemia, mother-to-foetus infection, and abortion [5]. There are several virulence genes so far identified in* L. monocytogenes*. These include the internalins (encoded by* inl* A,* inl* C, and* inl* J), listeriolysin O (LLO encoded by* hly* A), actin (*act* A), phosphatidylinositol-phospholipase C (PI-PLC encoded by* plc* A),* iap* (invasion associated protein encoded by* iap*), and virulence regulator (encoded by* prf* A). These virulence factors play significant role in the bacterial pathogenicity and infection outcome [6, 7].

It has been observed that* Listeria monocytogenes* employ these virulence factors that act synergistically in the intracellular pathogenicity of the bacteria [8]. For example, expressions of virulence genes, such as listeriolysin O (*LLO*), phospholipases (*plc A *and* plc B*), and internalins A and B (*inl A *and* inl B*), are regulated by* prf* A, and these genes facilitate the intracellular growth and spread of the bacterium within the mammalian host [9]. Point mutation is responsible for low virulence of* Listeria monocytogenes* strains in a number of virulence genes and as a result of spontaneous mutations, some* L. monocytogenes* strains may lose one or more virulence determinants [10, 11]. A virulence factor of phospholipase, called PI-PLC, is expressed by pathogenic species of* L. monocytogenes* and* Listeria ivanovii* only, and this virulence factor is an important marker for the discrimination between pathogenic and nonpathogenic* Listeria* species [12]. There is growing evidence that virulence is not a stable property but can be influenced by environmental conditions. For example, it has been shown that acid and salt stress increase the expression of virulence genes and* in vitro *pathogenicity of the bacteria [13]. Virulence potentials are also influenced by temperature, the presence or absence of oxygen, osmotic stress, and pH [14].* L. monocytogenes* has 13 serotypes, of these only four serotypes (1/2a, 1/2b, 1/2c, and 4b) are known to cause human listeriosis [15]. Based on the data generated from molecular subtyping methods* L. monocytogenes* isolates have been grouped into two major genetic divisions or lineages, termed as lineage I and lineage II [15]. A third lineage, predominantly associated with animals, has been described by some studies as well [16]. Serotypes 1/2a, 3b, 3c, and 4b belong to lineage I, while serotypes 1/2a, 1/2c, and 3a belong to lineage II [17]. Studies have shown that lineage I strains are significantly higher among human clinical listeriosis cases and contaminated foods [1], while lineage II strains show a significantly higher prevalence among food isolates and animal clinical cases than among human clinical listeriosis [16]. Pathogenic potentials of lineage I isolates are greater than those of lineage II, as determined by their ability to spread to neighbouring host cells in a cell culture plaque assay [1]. It will be observed that there is a consensus that lineage I strains may represent a human host-adaptive lineage while lineage II strains may represent an environmental adapted lineage [15]. The application of multiplex PCR for the detection of more than one virulence gene in a single tube is desirable because it reduces cost and labour and will be useful in a large scale detection of virulent strains of* Listeria* [18].

The prevalence and molecular characteristics of* Listeria monocytogenes* are generally unknown in Nigeria and thus this present study was carried out with the aim of characterizing* L. monocytogenes* isolates from raw milk and milk products using PCR and to determine the virulence and the phylogenetic characteristics of the isolates.

## 2. Materials and Methods

### 2.1. Sample Collection

#### 2.1.1. Bacterial Strains and Biochemical

The isolation of* Listeria monocytogenes* was carried out according to the procedure of Roberts et al. [19]. Briefly, about 10 mL of the incubated homogenate was added to 90 mL* Listeria* enrichment broth (Oxoid, CM 0862), which contains selective* Listeria* enrichment supplements (Oxoid, SR 0141E) and incubated at 30°C for 48 hrs. A loop full of the 48 hrs broth was cultured onto Chromogenic* Listeria* agar (Oxoid, CM 1080) plates, which contains brilliance*™ Listeria* differential supplement (Oxoid, SR 0228E) and brilliance*™ Listeria* selective supplement (Oxoid, SR 00227) and the plates was incubated at 37°C for 24–48 hrs. Colonies of* Listeria monocytogenes* having a greenish-blue coloration were picked and streaked on nutrient agar (Oxoid, CM 0003) slants and incubated at 37°C for 24 hrs, before storage at 4-5°C. The suspected isolates were further subjected to conventional biochemical test [Gram staining, oxidase, catalase, *β*-hemolysis, bile esculin, and carbohydrate fermentation test (xylose, Mannitol, Rhamnose, Maltose, Inositol, and Sucrose)] as described previously [20]. Isolates found to ferment mannitol and xylose and positive to rhamnose were considered as* L. monocytogenes. *Further characterization using Microbact*™* 12L* Listeria* identification kit (Oxoid, MB 1128) was carried out.

Following cultural identification and biochemical assays stated above 36* Listeria monocytogenes* isolates were identified (Table 1).

### 2.2. Polymerase Chain Reaction and Gel Electrophoresis

Bacterial isolates obtained locally as well as reference strains were subjected to PCR assay. The reference strain designated ATCC 19155 used as positive control was kindly provided by Professor Radu of the Center of Excellence for Food Safety Research, Faculty of Food Science and Technology, Universiti Putra Malaysia, where the molecular studies were carried out. Bacterial genomic DNA was extracted from both local and reference isolates using the Quick-g DNA*™* miniprep kit, (Zymo Research, SA) following the manufacturer's instructions.

Multiplex PCR assay was used to detect* L. monocytogenes* harbouring* hly* A gene and 16S rRNA of* L. monocytogenes *[21]. The primer pairs designated as LM1 and LM2 (LM1 5′–CCT AAG ACG CCA ATC GAA-3′ and LM2 – 5′-AAG CGC TTG CAA CTG CTC-3′) were used for the detection of* L. monocytogenes* harbouring* hly* A gene. On the other hand, primer pairs designated as U1 and LI1 (LI1 5′–CTC CAT AAA GGT GAC CCT-3′ and U1 5′–CAG CMG CCG CGG TAATWC-3′) were used to amplify 938 bp region in the 16S rRNA gene specific for the detection of* Listeria* genus [21]. These primers were synthesized by Invitrogen, USA, and were 18–20 bp in length.* L. monocytogenes* ATCC 19155 was used as a positive control.

The PCR amplification was carried out in a 25 *μ*L reaction mixture that consisted of 5 *μ*L of 5x PCR buffer, dNTPs (0.5 *μ*L), MgCl_2_ (2 *μ*L), Taq DNA polymerase (0.5 *μ*L), 0.5 *μ*L of each 20 pM primer [LM1, LM2, U1, and LI1], 13 *μ*L of distilled water, and 2 *μ*L of DNA template. PCR amplification was carried out in a programmed thermocycler with the following thermal conditions: hot start PCR plate at 95°C for 3 mins, followed by 35 cycles each of 30 sec denaturation at 94°C, 15 sec annealing at 53°C, 90 sec extension for 72°C, and final extension at 72°C for 7 mins.

Following PCR amplification, about 5 *μ*L of the PCR product was mixed with DNA loading dye (6x) and electrophoresed in 1.0% Agarose gel in TAE buffer using a minitank at 80 V, 400 amp, and 45 min. The electrophoresed product on the gel was stained with ethidium bromide for 30 min, destained for 20 min, and visualized under UV illuminator (SYNGENE, Biosystems, UK). A 100 bp DNA ladder (Promega Corporation, USA) was included to estimate the size of the amplified products.

### 2.3. Detection of* L. monocytogenes* Virulence-Associated Genes

The obtained DNA product was used in performing another multiplex PCR assay with the aim of detecting the presence of five virulence-associated genes [hemolysin A gene (*hly *A), regulatory gene (*prf *A), actin gene (*act *A), invasion associated protein p60 gene (*iap*), and surface protein (*inl* A)] of* L. monocytogenes,* as described by Rawool et al. [18]. The primers used for the detection of these virulence genes were synthesized by Sigma Aldrich, USA. The details of the primer sequences are shown in Table 2. The DNA obtained using the Quick-g DNA miniprep-uncapped column kit (Zymo Research) was used for the detection of virulence-associated genes.

The PCR was standardized for the detection of virulence-associated genes of* L. monocytogenes* by optimizing the annealing temperatures between 50 and 60°C. Based on optimization trials, the standardized PCR protocol for a 25 *μ*L reaction mixture included 5 *μ*L PCR buffer (100 mMTris–HCl buffer, pH 8.3 containing 500 mM KCl, 15 mM MgCl_2_, and 0.01% gelatine), 1 *μ*L dNTP mix, 3 *μ*L MgCl_2_ and 0.5 *μ*L of forward and reverse primers, 1 *μ*L of 5 units of Taq DNA polymerase, 5 *μ*L DNA template, and 5 *μ*L nuclease free water to make up the reaction volume. The DNA amplification reaction was performed in a Master Cycler gradient thermocycler (Eppendorf, Hamburg, Germany) with a preheated lid in PCR tubes (0.2 mL). The cycling conditions for PCR included an initial denaturation of DNA at 95°C for 2 min followed by 35 cycles each of 15 sec denaturation at 95°C, 30 sec annealing at 53°C, and 90 sec extension at 72°C. Final was carried out in 10 min at 72°C and then PCR product held at 4°C.

The resultant PCR products were separated by electrophoresis in 1.5% Agarose gel for 45 min at 100 V in Tris-acetate EDTA buffer, stained with ethidium bromide (0.5 *μ*g/mL), and visualized with a UV transilluminator (Syngene Frederick, MD). The gel image was documented by a gel documentation apparatus, and DNA size was determined by a 100 bp DNA ladder (Promega).

### 2.4. Purification of PCR Products

5 *μ*L volume PCR product for each of the 9* hly* A and 16S rRNA harbouring* L. monocytogenes* positive isolates was analysed on a 1.5% Agarose gel to confirm successful amplification of the 16S rRNA. The remaining 20 *μ*L of each of the PCR product was later run and each corresponding 16S rRNA band was cut and purified by using the MEGA quick-spin*™* Total Fragment DNA purification kit (iNtRON Biotechnology, Gyeonggi-do, South Korea).

### 2.5. DNA Sequencing and Phylogenetic Analysis

Purified gel was used for DNA sequencing. All sequencing reactions were commercially done by iNtRON (iNtRON Biotechnology Gyeonggi-do, South Korea) using the Sangers sequencing method. Both forward and reverse primers were used to ensure adequate sequencing coverage. For sequencing and phylogenetic tree, only 15 samples were randomly selected and tested.

The obtained partial 16S rRNA sequences were proofread and assembled in Chromas Lite version 2.1.1. Multiple sequence alignment of the DNA sequences was performed using ClustalW in Bioedit software version 7.2.5 (Biological Sequence Alignment Editor for Win 95/98NT/2 K/XP/7), as described by Hall, 1999 [22]. To compare the sequence of local* L. monocytogenes* with reference isolates available in the GenBank database, sequence similarity search was performed using the NCBI nucleotide BLAST search to determine the similarity of the local* L. monocytogenes *sequence (http://blast.ncbi.nlm.nih.gov/).

Phylogenetic analysis was carried out based on partial sequence of 16S rRNA gene of* L. monocytogenes*. In this regard, the aligned sequence was subjected to a Maximum Likelihood (ML) method, in MEGA 6.0 software [23]. However, prior to performing ML analysis, best substitution model that described the sequence data set was obtained taking into considerations the lowest Bayesian inferences. Equally, 1000 bootstraps values were used to determine the confidence interval of the resultant tree. In order to root the tree, a 16S rRNA sequence from* Mycobacterium tuberculosis* was used as an out-group.

All local* L. monocytogenes* sequences used in the phylogenetic study were submitted to GenBank for archiving. The accession numbers of these sequences together with the reference isolates are summarised in Table 3.

## 3. Result

### 3.1. Detection of* hly* A Harbouring* Listeria monocytogenes *Isolates

**Figure 1:**
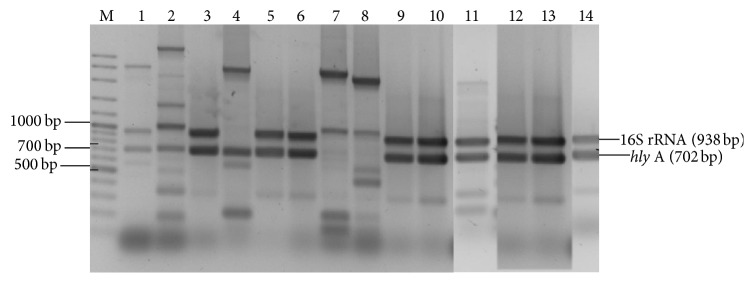
Agarose gel electrophoresis showing electrophoretic mobility of PCR products obtained following amplification of the 16S rRNA gene (938 bp) and listeriolysin O encoding gene (*hly* A) (702 bp) from different* Listeria monocytogenes* isolated from raw milk and milk products. Lane M: 100 bp ladder; Lane 1: positive control (*Listeria monocytogenes* ATCC 19155); Lane 3:* Listeria monocytogenes* (LM) isolate ZKMM84; Lane 5: LM SGHR 30; Lane 6: LM SGSK1; Lane 9: LM ZKJR56; Lane 10: LM SGHR39; Lane 11: LM ZKMR75; Lane 12: LM SGMM87; Lane 13: LM SGHR15; Lane 14: LM LMFR162, corresponding to 938 bp and 702 bp amplicons.

The PCR results for the detection of* hly *A gene among the 36* L. monocytogenes* isolates showed that only 9 (25.0%) (ZKMM84, SGHR30, SGSK1, ZKJR56, ZKJR39, ZKMR75, SGMM87, SGHR15, and LMFR162)* L. monocytogenes* isolates harboured the* hly *A gene (Figure 1).

### 3.2. Detection of Virulence-Associated Genes of* Listeria monocytogenes *Isolates

**Figure 2:**
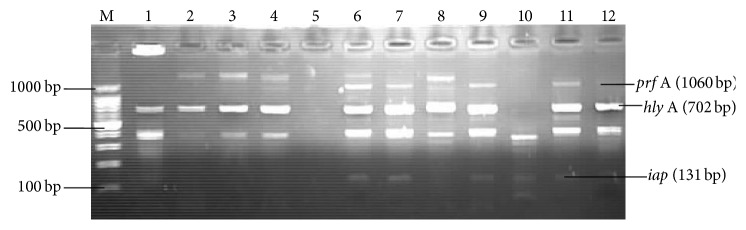
Agarose gel electrophoresis result for multiplex PCR following amplification of* prf* A,* hly* A, and* iap* virulence-associated genes. Lane M: molecular weight marker (Promega) in base pairs; Lane 1:* Listeria monocytogenes* (LM) ATTC 19155; Lane 2: LM isolate ZKMM84; Lane 3: LM SGHRR30; Lane 4: LM SGSK1; Lane 5: negative control; Lane 6: LM ZKJR55; Lane 7: LM SGHR39; Lane 8: LM ZKMR75,=; Lane 9: LM SGMM37; Lane 11: LM SGHR15; Lane 12: LMFR162.

Analysis of multiplex PCR used for the detection of virulence-associated genes revealed that, of the 36* L. monocytogenes* isolates, 9 (25.0%) possessed at least one or more virulence-associated genes (Figure 2). Of the 5 virulence-associated genes screened for only 3 virulence-associated genes (*hly* A,* iap*, and* prf* A) genes were detected; each of 5 (13.8%) isolates possessed the* iap* and* prf *A genes. Based on the sample types, 6 (16.9%) of the* L. monocytogenes *isolates that possessed virulence-associated genes were from raw milk, 2 (3.2%) “Manshanu,” and 1 (2.8%) “Kindrimo”.

### 3.3. Phylogenetic Tree Analysis

**Figure 3:**
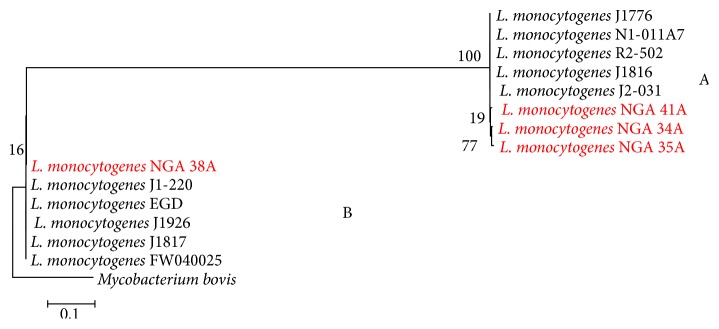
Molecular phylogenetic tree showing relationship between Nigerian* L. monocytogenes* isolates (red color) with reference sequences from GenBank database. The evolutionary history was inferred by using the Maximum Likelihood method based on the Tamura 3-parameter model. The tree with the highest log likelihood (−2760.2483) is shown. The percentage of trees in which the associated taxa clustered together is shown next to the branch and 1000 bootstrap value was used to assess the tree confidence interval. All analyses were performed in MEGA6 software.

Phylogenetic analysis was carried out based on the 16S rRNA sequences encompassing 938 bp obtained for 4 of the* L. monocytogenes* isolates (Nigerian isolates). Interestingly, maximum likelihood (ML) analysis revealed two different clusters A and B with cluster A comprising of Nigeria* L. monocytogenes *NGA 35A, NGA 34A, and NGA 41A and reference* L. monocytogenes *J1776; N1-011A; R2-502; J1816; and J2-031, while cluster B is comprised of Nigerian* L. monocytogenes* sequence NGA 38A and reference* L. monocytogenes* sequence EDG; J1-220; J1926; J1817; and FW040025. Sequence in cluster A was supported by a strong bootstrap values of 100 in contrast with cluster B which shows a very low bootstrap value of 16% (Figure 3).

## 4. Discussion

To ensure food quality and safety, detection of pathogenic bacteria should be a fundamental objective. The application of molecular techniques has facilitated the identification and characterization of major virulence-associated genes in* L. monocytogenes *[24]. In this study the multiplex PCR identified 9* Listeria monocytogenes* isolates harbouring* hly* A gene. The target genes for* L. monocytogenes* and* Listeria* genus produced PCR products of 702 bp and 938 bp in size, respectively. The PCR protocol used in this study was based on the amplification of* hly* A gene by using a set of primers, LM1 and LM2. Border et al. [21] recommended LM1 and LM2 primers as the best primer pair to be used for the detection of* L. monocytogenes*, because they are designed to amplify specific fragments in* L. monocytogenes* strains that are genetically and biochemically assessed as belonging to this specie, on the basis of the presence of the 702 bp and 938 bp amplicons [21].

Kuan et al. [25] were also able to amplify the DNA at their expected amplification site. The result of this study is not in agreement with the result of Jallewar et al. [26], who reported the carriage of* hly* A gene by all the isolates of* L. monocytogenes* tested. The inability of some of the* L. monocytogenes* isolates in this study to harbour the* hly* A gene may be due to the nonvirulent characteristics of some* L. monocytogenes* isolates because some may be environmental isolates or it may be possible that some* L. monocytogenes* strains may lack one or more virulence determinants because of spontaneous mutations [11].

The ability to survive and multiply in the macrophages and other host cells is key to the pathogenicity of* L. monocytogenes* [27]. Rapid methods for the isolation and confirmation of* L. monocytogenes* in foods are still being explored [28]. Because of the importance of* L. monocytogenes* epidemiology to human health and the notable diversity in the pathogenicity among strains, subtyping and virulence characterization are of importance [7]. Due to the diverse strains of* L. monocytogenes*, of varying pathogenicity, the ability to precisely track the strains involved in listeriosis outbreaks and rapidly determine their pathogenic potentials is critical for the control and prevention of further occurrences of this deadly disease [24]. The phenotypic subtyping methods are generally less sensitive, have low differentiation ability, and are not easy to reproduce, whereas the genotypic approaches are more sensitive and reliable [29]. Virulence-associated genes of* L. monocytogenes,* namely,* prf* A*, hly *A, and* iap*, were amplified according to their respective fragment sizes of 1060 bp, 702 bp, and 131 bp, respectively. This study revealed that of the 36* L. monocytogenes* isolates screened for virulence-associated genes, only 9 (25.0%) isolates harboured one or more of such genes and none of them possessed all the five genes. It is plausible that some* L. monocytogenes* strains may lack one or more virulence determinants because of spontaneous mutations [11].

Henriques et al. [30] detected* hly *A genes from* L. monocytogenes* isolates from Portuguese ready-to-eat meat based food industry, which is similar to the findings of this study where* hly* A was detected in all the nine* L. monocytogenes* isolates. However, the detection of* act* A gene in the work of Henriques et al. [30] differs from this study which failed to detect* act* A. Listeriolysin O and* act* A are associated with the bacterium's capability of passing the intestinal barrier, cell to cell spread and motility, cell invasion, and intracellular parasitism [7, 18]. Therefore, simultaneous detection of virulence genes in a single step will be desirable as it reduces time and labour, and it will be useful in large scale survey for detecting virulent strains of* Listeria *[18].

The separation of Nigerian* Listeria monocytogenes* isolates sequence (NGA 34A, NGA 35A, NGA 38, and NGA 41A) as well as the* L. monocytogenes* sequences from the GenBank (N1-011A, R2-502, J1816, J2-031, EDG, J1-220, J1926, J1817, and PW040025), into two major lineages, A and B, is consistent with the major lineages described previously by Oris et al. [31], who reported that* L. monocytogenes* isolates.

*L. monocytogenes* consist of at least 4 evolutionary lineages (I, II, III, and IV). Even though only cluster A is statistically supported with a bootstrap value of 100%, cluster B is not statistically supported.

Most* L. monocytogenes* isolates seem to belong to lineages I and II, which include the serotypes more commonly associated with human clinical cases, including serotypes 1/2b and 4b (lineage I) and serotype 1/2a (lineage II). Since it is a rooted tree, we can infer evolutionary relationship, information regarding the common ancestors, and the path of evolution from the rooted phylogenetic tree.

Since lineage I* L. monocytogenes* strains are more prevalent in human clinical listeriosis cases and contaminated food [31], it can be said that Nigerian* L. monocytogenes* isolates (NGA 34A, NGA 35A, and NGA41A) are from contaminated foods (i.e., milk and milk products). From the phylogenic tree, Nigerian* L. monocytogenes* isolates (NGA 34A, NGA 35A, and NGA 41A) were classified as lineage I, which are known to be of higher pathogenic potentials due to intrinsic virulence features, as compared to lineage II strains of* L. monocytogenes* [32]. Morobe et al. [33] in their study observed that most of the* L. monocytogenes* isolates belong to lineage III (4a and 4c), which are known to be more prevalent in animals with clinical listeriosis [31], as opposed to this study where none of the* L. monocytogenes* isolate were found to belong to lineage III. Isolate NGA 35a was found to belong to lineage II, which is opposed to the findings of Morobe et al. [33], where they observed that none of the* L. monocytogenes* isolates belong to lineage II.

The implication of the present findings is that* L. monocytogenes* of lineage 1 originating from animal milk and milk product could be associated with increased human risk to* L. monocytogenes* infection in Nigeria and thus effort to control the disease should be targeted at improving the food safety and quality.

## Figures and Tables

**Table 1:** *Listeria monocytogenes* characteristics.

S/number	Isolate name (code)	Isolated from	Tip name in tree
(1)	LMFR151	Raw milk	49A
(2)	ZKMM83	Manshanu	48A
(3)	MDGR115	Raw milk	47A
(4)	MMM103	Manshanu	46A
(5)	ZKMM84	Manshanu	34A
(6)	LSNK136	Kindrimo	44A
(7)	SGMK77	Kindrimo	43A
(8)	MGDR100	Raw milk	42A
(9)	ZKMR75	Raw milk	35A
(10)	SGHR39	Raw milk	38A
(11)	ZKJR55	Raw milk	40A
(12)	SGHR7	Raw milk	39A
(13)	SGHR12	Raw milk	37A
(14)	SGHR30	Raw milk	41A
(15)	ZKMM87	Manshanu	36A
(16)	ZKMM84	Manshanu	45A
(17)	ZKJR5	Raw milk	33A
(18)	SGMM14	Manshanu	32A
(19)	SGMM34	Manshanu	31A
(20)	SGHR45	Raw milk	30A
(21)	LMFR161	Raw milk	29A
(22)	MDGR109	Raw milk	28A
(23)	SGHR32	Raw milk	27A
(24)	MDGR10	Raw milk	25A
(25)	ZKJR10	Raw milk	24A
(26)	SGSM9	Manshanu	23A
(27)	LMFR162	Raw milk	15A
(28)	SGHR55	Raw milk	22A
(29)	MDGM123	Manshanu	21A
(30)	SGSK1	Kindrimo	26A
(31)	SGHR15	Raw milk	20A
(32)	SGHR37	Raw milk	19A
(33)	SGSK21	Kindrimo	18A
(34)	LMFK153	Kindrimo	17A
(35)	SGMM37	Manshanu	16A
(36)	SGMM11	Manshanu	14A

**Table 2:** Primer sequences used for the amplification of virulence-associated genes of *Listeria monocytogenes*.

Target gene	Primer sequence (5′-3′)	Product size (bp)
*inl *A	Fwd: 5′-AGATCTAGACCAAGTTACAACGCTTCAG-3′ Rev: 5′-TAA TAT CAT TTG CTG TTT TAT CTG TC-3′	255 bp

*act *A	Fwd: 5′′-ACG TGA AGT AAG TCACGT GAT ATT G 3′ Fwd: 5′′-ACG TGAAGTAAGCTCACGT GAT ATT G-3′	268 bp

*prf *A	Fwd: 5′-ACC GCT CAG AAA AGT TCT TC-3′ Rev: 5′-TCT TGT TCT ATT ATGTCT AGC-3′	1060 bp

*iap*	Fwd: 5′-ACA AGC TGC ACC TGT TGC AG-3′ Rev: 5-TGA CAG CGT TGT TAG TAG CA-3′	131 bp

*hly *A	Fwd: 5′-CCT AAG ACG CCA ATC GAA-3′ Rev: 5′-AAG CGC TTG CAA GTC CTC-3′	702 bp

**Table 3:** Local and reference *L. monocytogenes* isolates used in the phylogenetic analysis.

S/number	Strain	GenBank accession number
(1)	*Listeria monocytogenes* NGA 34A	KX358070
(2)	*Listeria monocytogenes* NGA 35A	KX358071
(3)	*Listeria monocytogenes* NGA 38A	KX358072
(4)	*Listeria monocytogenes* NGA 41A	KX358073
(5)	*Listeria monocytogenes* N1-011A	CP00659
(6)	*Listeria monocytogenes* J1816	CP006047
(7)	*Listeria monocytogenes J2-031*	CP006593
(8)	*Listeria monocytogenes J1-220*	CP0060461
(9)	*Listeria monocytogenes J1926*	CP006600
(10)	*Listeria monocytogenes J1817*	CP006599
(11)	*Listeria monocytogenes R2-502*	CP006594
(12)	*Listeria monocytogenes J1776*	CP006598
(13)	*Listeria monocytogenes EDG*	HG421741
(14)	*Listeria monocytogenes FW040025*	CP011345
(15)	*Mycobacterium bovis*	AY360331
